# A High Index of Awareness About the Inherent Complications of Thoracic Segmental Spinal Anesthesia: A Case of Mastectomy With Bronchiectasis Under Thoracic Segmental Spinal Anesthesia

**DOI:** 10.7759/cureus.76586

**Published:** 2024-12-29

**Authors:** Kirti Gujarkar Mahatme, Pratibha U Deshmukh, Anjali Borkar, Nandkishor J Bankar, Prajwal Mahatme

**Affiliations:** 1 Anaesthesiology, Datta Meghe Medical College, Datta Meghe Institute of Higher Education and Research (Deemed to be University), Nagpur, IND; 2 Microbiology, Jawaharlal Nehru Medical College, Datta Meghe Institute of Higher Education and Research (Deemed to be University), Wardha, IND; 3 Urology, Datta Meghe Medical College, Datta Meghe Institute of Higher Education and Research (Deemed to be University), Nagpur, IND

**Keywords:** case report, mastectomy, segmental spinal, spinal sedation, thoracic, thoracic segmental spinal

## Abstract

General anesthesia is the gold standard for breast cancer surgeries. Considering the nature of the surgery and its associated pain, various regional techniques are used as an adjunct to general anesthesia. Regional anesthesia as a sole anesthetic technique for breast cancer surgery is an upcoming technique - especially in high-risk patients considering the risk-benefit ratio, various regional blocks like pectoralis major block, pectoralis minor block, and erector spinae block - in which thoracic segmental spinal anesthesia is the recent one. Here we present a 68-year-old patient with bronchiectasis for radical mastectomy under thoracic segmental spinal anesthesia with isobaric bupivacaine at T4-5 intervertebral space, achieving blockade at T1 to T8 level. The patient complained of respiratory distress and hoarseness of voice after 40 minutes of surgery, which was successfully managed. Early recognition and timely management of the untoward effect helped us to complete the case uneventfully. In this article, we emphasize that patient safety and selection of the type of anesthesia are of utmost importance, and hoarseness of voice and sedation caused due to the adjuvant added intrathecally should always be considered alarming sign during a thoracic segmental spinal anesthesia as well as conventional lumbar spinal anesthesia.

## Introduction

Though general anesthesia is considered to be the gold standard for breast surgery, it is substantially associated with complications in geriatric patients, thereby increasing the risk of morbidity and mortality. Regional anesthesia can be used as an alternative technique where complications with general anesthesia are anticipated [1]. We performed a radical mastectomy in a 68-year-old female with bronchiectasis under thoracic segmental spinal anesthesia. The induction and maintenance of the case were smooth until an event occurred, which was timely managed to complete the case successfully. In the era of evidence-based medicine, there are multiple studies which have proven the safety of thoracic segmental spinal anesthesia and thus the surgeries which were thought once only under general anesthesia are successfully done under segmental spinal anesthesia [2].

## Case presentation

A 68-year-old female diagnosed with high-grade intraductal breast carcinoma was admitted for radical mastectomy and axillary lymph node dissection. She was a known case of bronchiectasis with chronic obstructive respiratory disease and cardiac function with concentric hypertrophy. Thus, the surgery was planned under regional anesthesia (thoracic segmental spinal anesthesia), considering the risk-benefit ratio of general anesthesia.

**Figure 1:**
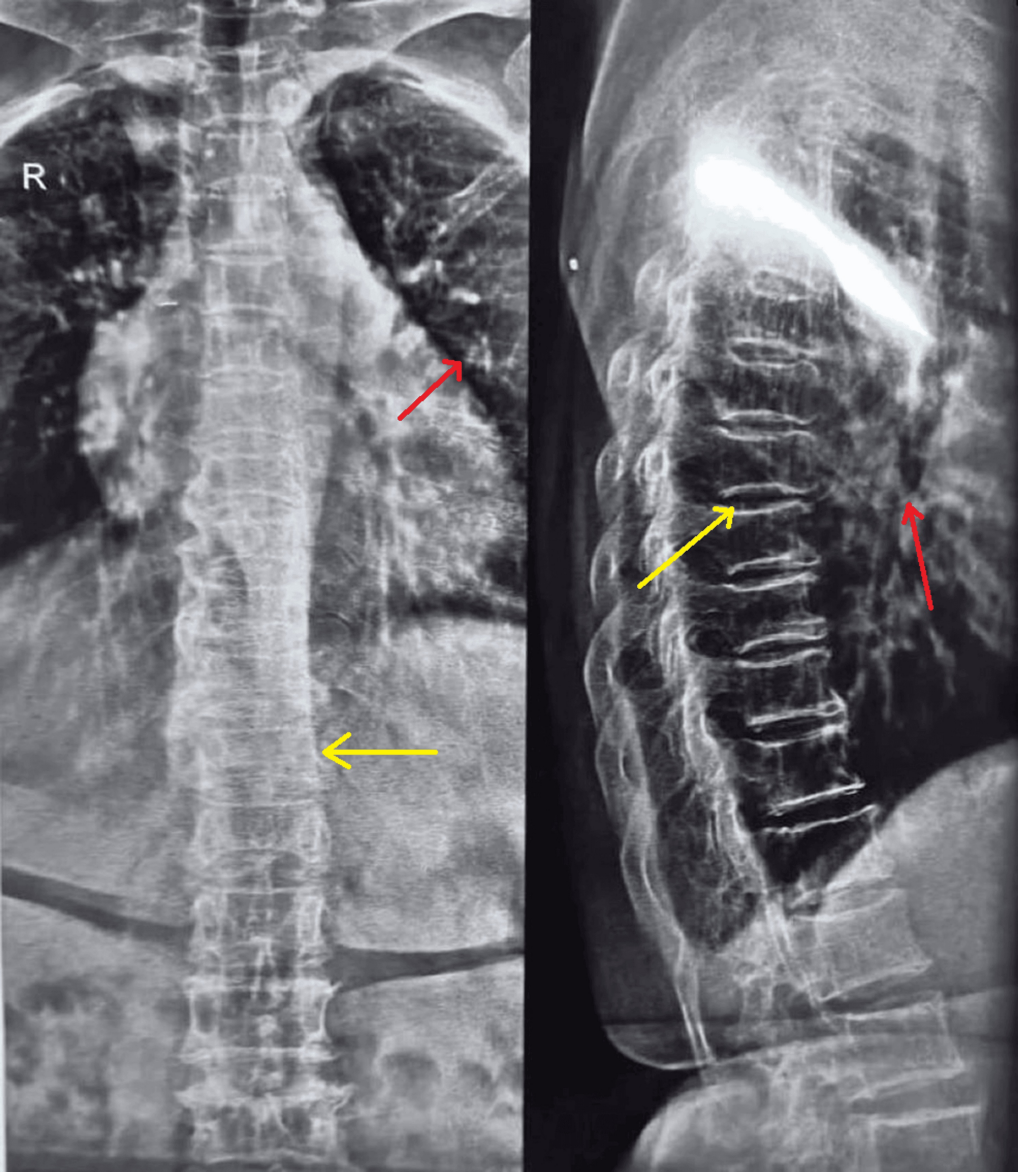
X-ray showing kyphoscoliotic changes in the spine and chronic obstructive pulmonary disease (COPD) changes in the lungs Red arrows showing chronic obstructive pulmonary disease lung; Yellow arrows showing kyphoscoliotic spine.

A pre-anesthetic checkup was done, and routine investigations like clinical blood count electrolytes, kidney function test, electrocardiogram (ECG), and two-dimensional (2D) echo were advised, including a computer tomography (CT) scan of the chest to see the severity of bronchiectasis and a respiratory physician opinion was taken (Figure 1).

Written informed consent was taken explaining the risk and type of anesthesia under the American Society of Anaesthesiology physical status III. The patient was informed to stay nil per oral for six hours before surgery and to continue nebulization as advised by the respiratory physician. The patient was shifted to the operation theatre. A wide bore intravenous line of 18 gauge was secured on the right upper extremity that is opposite to the operative site. All five ASA I monitors were attached, and vitals were checked - heart rate of 86/min, blood pressure of 144/88 mmHg, a saturation of 92% in the room air, with oxygen at two liters maintaining 96%. Injection hydrocortisone 100 mg and injection ondansetron 4 mg were given intravenously. Thoracic segmental spinal anesthesia was performed in the right lateral position under all aseptic precautions at T4-5 interspace after infiltration with 1 ml of plain lignocaine 2% as a local anesthetic. Using a 25G quincke spinal needle, a subarachnoid block was given with a combination of 1 ml of 0.5% isobaric bupivacaine and 25 mcg of fentanyl after confirming the free flow of clear cerebrospinal fluid. The patient was turned to the supine position immediately, and the oxygen mask was attached. The patient was vigilantly monitored vitally till the required sensory and motor blockade was achieved at T1-8 level within 8 minutes of performing the regional block. Injection glycopyrrolate, injection atropine, and injection mephentermine were kept loaded and handy in case needed for bradycardia or hypotension. T1-T8 was completed within 8 minutes of the block, and the case was started.

The vitals were very stable when suddenly, after 50 minutes, the patient complained of hoarseness of voice and was counseled and cautiously monitored. There we noticed some breathing difficulties for the patient, and within 5 minutes, patient saturation dropped from 96% to 92% to 85% to 70%, and heart rate dropped to 49/min. Immediately oxygen mask was held, and an injection of atropine 0.6 mg was given. We planned to intubate the patient without any delay. The patient’s saturation increased, and he was intubated with cuffed endotracheal tube (ETT) number seven. After bilateral air entry was checked, the tube was fixed to 20. After that, the surgeon continued surgery for the next two hours. But to our surprise, the patient didn't require anesthesia to maintain the tube. Just oxygen was kept with air at two liters. The patient was awake, cooperative, and following commands. At the end of the surgery, the patient started moving his hands and legs, for which we had to start sevoflurane for the last 10 minutes. After surgery, the patient was extubated, driving all four limbs and following commands. The patient was observed for two hours post-op recovery and followed up for three days for any other neurological deficit.

The thoracic segmental spinal anesthesia plan was executed well in the patient at the T4-5 level. The level of T1-T8 was achieved within 8 minutes. The patient complained of hoarseness of voice after 50 minutes. The patient got restless and complained of breathing difficulty at 60 minutes; thus, oxygen flow was increased to six liters. At 60 minutes, SpO2 suddenly started dropping to 70%, and the heart rate dropped to 45/min. The mask with circuit was held with 100% oxygen with which saturation started to increase. Injection atropine 0.6 mcg was given to treat bradycardia. At 60 minutes, the patient was intubated, and after getting vitally stable, surgery was continued. The patient was maintained on oxygen and air; no other anesthetic agents were required to maintain the tube for the patient. Surgery was continued for two hours. After three hours, the patient was extubated, conscious, following commands, and vitally stable with a heart rate of 82/min, blood pressure (BP) of 132/76 mmHg, and maintaining saturation of 100%.

## Discussion

Oncological surgeries should be prioritized as delay in operating may worsen cancer staging. However, giving general anesthesia to patients with cardiorespiratory disease may produce detrimental results. Thus at such times, regional anesthesia plays a significant role. Various studies have been done by Paliwal et al., van Zundert et al., and Khan et al. on thoracic segmental spinal anesthesia as an alternative for surgeries like lap cholecystectomy, mastectomy where general anesthesia is ideal and found to have comparable results and surgeon satisfaction [3-6].

Thoracic spinal anesthesia is a century-old technique that was practiced with hyperbaric bupivacaine earlier. However, recently isobaric drugs have been introduced due to their several advantages such as it spreads to the limited area where the drug is injected, thus preventing the mishap of threatening complication known as total spinal anesthesia. Dr Len Carrie has shown the spread of isobaric bupivacaine in a glass model of the human spine. Imbelloni et al. have conducted a study to demonstrate the safety of thoracic segmental spinal anesthesia using an MRI to evaluate the spread of the drug [7,8]. But even after all these precautionary measures, there may be some problems. Our case report emphasizes that the possible cause of hypotension and bradycardia may be the sedation caused due to the adjuvant added intrathecally to the spinal drug to prolong the action during spinal anesthesia. Though the protein drug and dose were given to the patient, and the case was stable for almost an hour, the effect of spinal sedation can occur after a speculated time. Intrathecal fentanyl follows a three-compartment model of drug distribution: primary distribution time is 1.7 minutes, redistribution time is 13 minutes, and terminal elimination time is 219 minutes. The half-life of intrathecal fentanyl is almost 3.5 hrs, and delayed respiratory depression can occur till 6-10 hrs.

The systematic review conducted by Karim et al. [9] highlights that while segmental thoracic spinal anesthesia (STSA) is associated with significantly higher odds of hypotension and bradycardia compared to general anesthesia (GA), it demonstrates clear advantages in terms of superior postoperative pain control, reduced opioid consumption, shorter post-anesthesia care unit (PACU) stays, and significantly lower incidence of postoperative nausea vomiting (PONV), indicating its potential as a viable alternative to GA for upper abdominal and thoracic surgeries in selected patient populations.

Our article aims to make people aware of the continuous monitoring of spinal sedation and its delayed effects. The paper mentions various segmental spinal complications, like direct needle trauma, infection, vertebral column hematoma, spinal cord ischemia, and total spinal anesthesia. Still, there is no mention of hoarseness of voice in any article. Thus, in our report, the hoarseness of the voice is also one of the complications.

## Conclusions

Regional anesthesia is acceptable for mastectomy considering the complications of general anesthesia. Hoarseness of voice is one of the complications we noticed; further studies are needed to confirm it. Spinal sedation should always be considered, and vigilant monitoring should be there throughout surgery as mishaps can occur at any time. This article emphasizes that patient safety and selection of the type of anesthesia are of utmost importance, and hoarseness of voice and sedation caused due to the adjuvant added intrathecally should always be considered alarming sign during thoracic segmental spinal procedures.
